# Effect of Curcumin on Hepatic mRNA and lncRNA Co-Expression in Heat-Stressed Laying Hens

**DOI:** 10.3390/ijms25105393

**Published:** 2024-05-15

**Authors:** Xinyue Wu, Xubin Du, Huifang Pian, Debing Yu

**Affiliations:** 1 Department of Animal Genetics, Breeding and Reproduction, College of Animal Science and Technology, Nanjing Agricultural University, Nanjing 210095, China; wxy18943109916@163.com (X.W.); 15666289565@163.com (H.P.); 2 Single Molecule Nanometry Laboratory (Sinmolab), Nanjing Agricultural University, Nanjing 210095, China; dxb950227@163.com

**Keywords:** curcumin, heat stress, laying hens, RNA-seq, lncRNA, transcriptome analysis

## Abstract

Heat stress is an important factor affecting poultry production; birds have a range of inflammatory reactions under high-temperature environments. Curcumin has anti-inflammatory and antioxidant effects. The purpose of this experiment was to investigate the effect of dietary curcumin supplementation on the liver transcriptome of laying hens under heat stress conditions. In the animal experiment, a total of 240 Hy-Line brown hens aged 280 days were divided randomly into four different experimental diets with four replicates, and each replicate consisted of 15 hens during a 42-D experiment. The ambient temperature was adjusted to 34 ± 2 °C for 8 h per day, transiting to a range of 22 °C to 28 °C for the remaining 16 h. In the previous study of our lab, it was found that supplemental 150 mg/kg curcumin can improve production performance, antioxidant enzyme activity, and immune function in laying hens under heat stress. To further investigate the regulatory mechanism of curcumin on heat stress-related genes, in total, six samples of three liver tissues from each of 0 mg/kg and 150 mg/kg curcumin test groups were collected for RNA-seq analysis. In the transcriptome analysis, we reported for the first time that the genes related to heat stress of mRNA, such as *HSPA8*, *HSPH1*, *HSPA2*, and *DNAJA4,* were co-expressed with lncRNA such as *XLOC010450*, *XLOC037987*, *XLOC053511*, *XLOC061207*, and *XLOC100318*, and all of these genes are shown to be down-regulated. These findings provide a scientific basis for the possible benefits of dietary curcumin addition in heat-stressed laying hens.

## 1. Introduction

Heat stress is the main factor affecting the production performance and health status of poultry; when the environmental temperature is too high, the physiological indicators such as poultry egg production performance, feed intake, egg quality, metabolism, and immune status will appear to be adverse changes [1]. High-temperature exposure led to free radical production, up-regulating macromolecular peroxidation, plasma superoxide dismutase, and glutathione peroxidase [2,3]. Heat stress causes oxidative damage to liver tissue, leading to liver injury and tissue damage [4].

Birds do not have sweat glands and dissipate heat mainly via air exchange and feather flapping [5]. Birds are extremely sensitive to variations in temperature; the most suitable environmental temperature for the production of adult laying hens is 19–22 °C, and whether it is more or less than this temperature needs to be thermoregulation [6]. Chronic heat stress reduces the expression of hepatic growth hormone mRNA as well as body weight gain, carcass yield, and serum antioxidant activity in broilers [7]. It was shown that the genes of the energy metabolism pathway in the liver and jejunal mucosa of broiler chickens were up-regulated under heat stress, and the oxidative stress of the organism was increased [8].

Curcumin possesses noteworthy anti-inflammatory and antioxidant properties, which render it efficacious in ameliorating a spectrum of maladies encompassing neurological disorders, cardiovascular ailments, and obesity [9,10]. Previous studies have found that adding appropriate amounts of curcumin to the diet of laying hens can alleviate the negative effects of heat stress and reduce the infiltration of inflammatory cells around the central hepatic vein and that curcumin can also improve the immune status of laying hens by decreasing the gene expression of TLR4 [11]. Moreover, the physiological stress and cardiac injury were attenuated in mice under heat stress by adding curcumin [12]. Previous studies conducted within our laboratory have elucidated that the addition of curcumin to the diets significantly enhances the egg production performance of heat-stressed laying hens [13]. However, the effect of curcumin on hepatic transcriptome expression in heat-stressed laying hens is also not known.

LncRNAs are a set of transcripts with important biological functions, and their aberrant expression has been associated with a variety of diseases and related mRNA disorder [14]. They are often defined as non-protein-coding transcripts that can be spliced, capped, and polyadenylated but had no sequence conservation, and lncRNA expression was lower and more tissue-specific than mRNA [15]. In the previous study, 156 up-regulated and 18 down-regulated differentially expressed lncRNAs were found in the bovine mammary gland under the heat stress condition [16]. It was reported that 174 up-regulated and 308 down-regulated differentially expressed lncRNA were identified in the liver of rats under heat stress, and the DElncRNAs associated with differentially expressed genes that encode heat shock proteins may play an essential role in heat stress response, including *HSF4*, *Dnaja1*, *Dnaja4*, *HSPH1*, and *HSPB1* in liver [17]. Therefore, we speculated that there are similar conditions of differentially expressed lncRNAs in hen’s liver under heat stress. The aim of this study was to reveal the effect of curcumin on hepatic lncRNA in heat-stressed laying hens.

## 2. Results

### 2.1. Quality Assessment of Sequencing Data

After quality control, 845,877,930 clean reads were obtained from 861,728,314 raw reads from six liver tissue samples. The clean reads of each sample were not less than 17G. The clean reads were determined by the quality score. The percentage of bases with quality scores greater than or equal to 30 was more than 92% of the total bases (Table 1). Data quality is excellent for subsequent analysis.

### 2.2. Reference Sequence Alignment and Splicing of Transcripts

**Figure 1:**
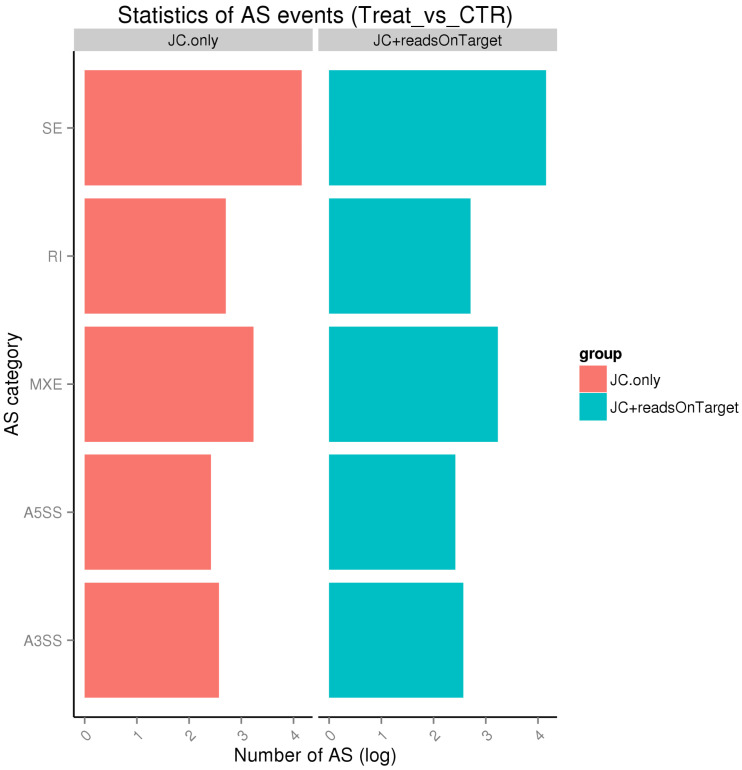
The typology and number of AS events. The *x*-axis stands for the typology of the AS event, SE is skipped exon, RI is retained intron, MXE is mutually exclusive exon, A5SS is alternative 5′ splice site, and A3SS is alternative 3′ splice site. The *y*-axis stands for the number of AS events.

HISAT2 (Version: 2.0.4) was used for mapping the filtered reads on the chicken reference genome (Gallus_gallus-4.0). The number of reads that can be aligned to the reference genome is more than 93%, and the number of reads that have a unique alignment position on the reference sequence is higher than 80% (Table 2). Alternative splicing analysis of the sequencing data with rMATS (Version: 3.2.5) resulted in the highest total number of AS events in exon jumps and the lowest total number of AS events in alternative 5’ splice site, whether using junction counts alone for AS event detection or both junction counts and reads on target for AS event detection (Figure 1). The above data shows that the comparison results are good and can be used for the next analysis.

### 2.3. The Result of lncRNA Filtration

**Figure 2:**
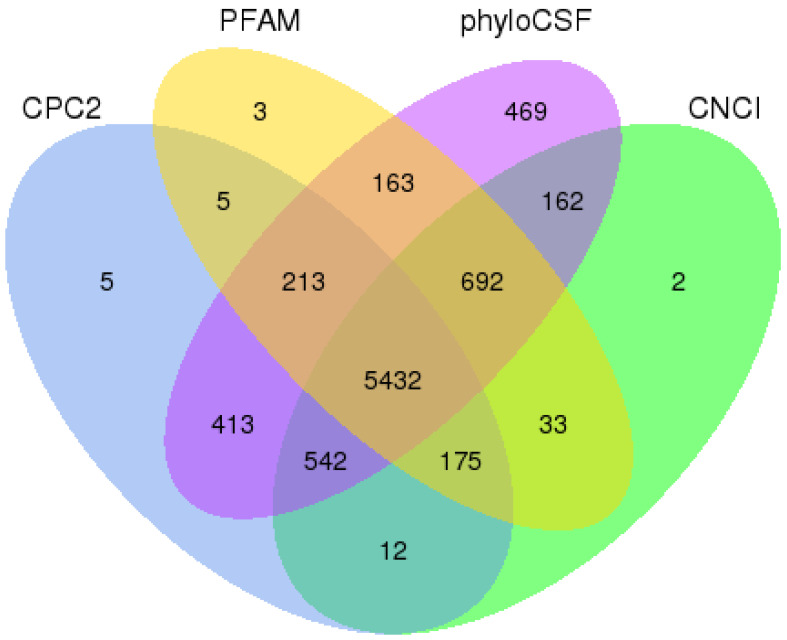
Venn diagram of lncRNA filtration. CPC2 is a Coding Potential Calculator. PFAM is the Protein Families Database. phyloCSF is a Phylogenetic Codon Substitution Frequencies. CNCI is the Coding-Non-Coding Index.

The number of lncRNA filtration using the cuffmerge program was consistent with the predictions of CPC2 (Coding Potential Calculator, Version: 2.0), PFAM (Protein Families Database, Version: 1.3), phyloCSF (Phylogenetic Codon Substitution Frequencies, Version: 1.0) and CNCI (Coding-Non-Coding Index, Version: 2.0), their predictions were 6797, 6716, 8086 and 7050. The intersection of the four software is 5432, which is consistent with the cuffmerge prediction (Figure 2).

### 2.4. Correlation Test between Samples

**Figure 3:**
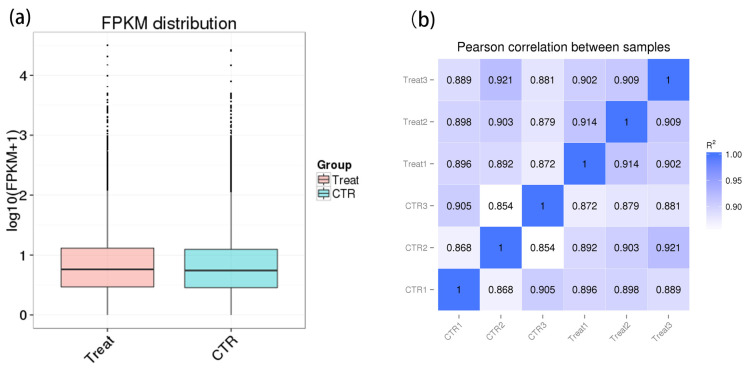
(**a**) Comparison of gene expression levels under the same experimental conditions; (**b**) the heatmap of the Correlation coefficient between samples.

As shown in Figure 3a, the distribution of duplicate samples is relatively close, suggesting that biological replicates are more successful. The Pearson correlation coefficient (*R*^2^) between samples was more than 0.8, indicating a high correlation between samples (Figure 3b). Therefore, the samples selected for this test met the sequencing requirements and can be analyzed in the next step.

### 2.5. The Differentially Expressed of lncRNA and mRNA

**Figure 4:**
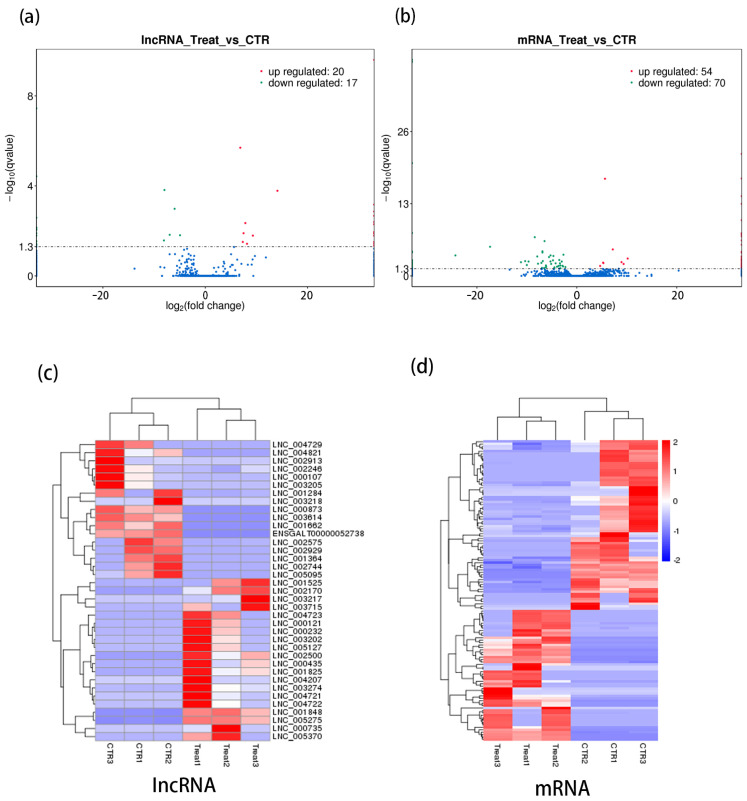
(**a**) Differentially expressed lncRNA volcano map; (**b**) Differentially expressed mRNA volcano map; (**c**) Differentially expressed lncRNA heat map; (**d**) Differentially expressed mRNA heat map.

For differential expression detection, the screening threshold was set to qvalue < 0.05 by default, and the screening yielded 20 up-regulated and 17 down-regulated differentially expressed lncRNAs (Figure 4a). Meanwhile, 54 of the differentially expressed mRNAs were screened for up-regulation and 70 for down-regulation (Figure 4b). The different expression patterns of lncRNAs and mRNAs in the control and experimental groups were evident from the clustering in the heatmap of differentially expressed lncRNAs and mRNAs (Figure 4c,d).

### 2.6. GO Enrichment Analysis

**Figure 5:**
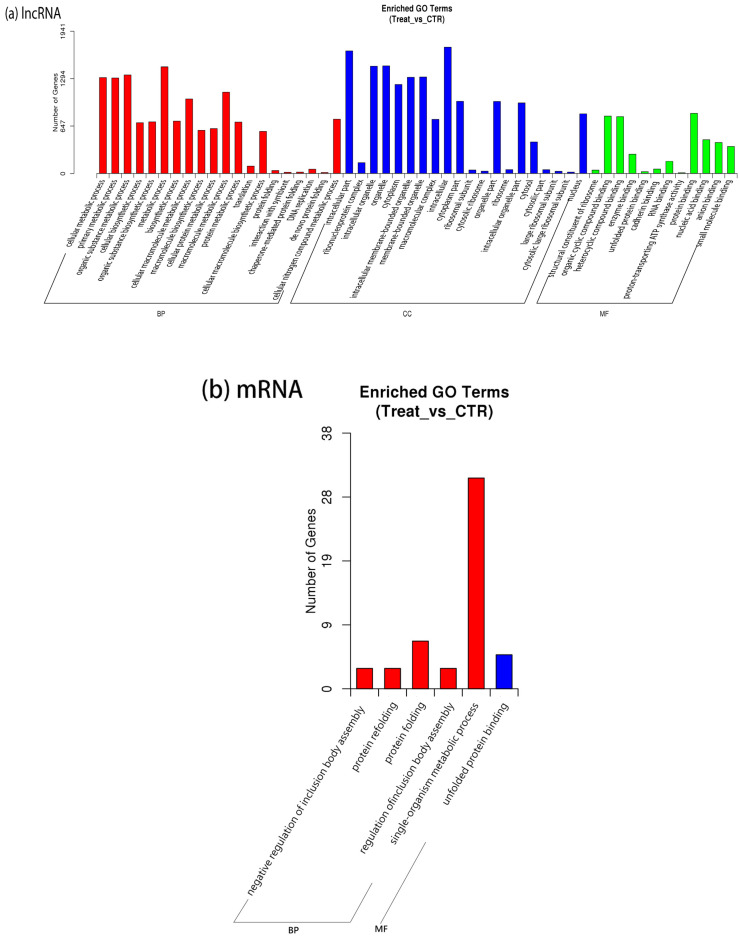
(**a**) Histogram of GO enrichment of differential expression of lncRNA target genes; (**b**) Histogram of GO enrichment of differential expression of mRNA target genes. The *y*-axis presents the number of genes. The *x*-axis presents the enriched GO terms. BP is biological processes, CC is cellular components, and MF is molecular functions.

The target genes of the screened lncRNAs and mRNAs were subjected to GO enrichment analysis, thereby predicting the primary functions of the differentially expressed lncRNAs and mRNAs, and the GO entries of the target genes were annotated at the levels of biological processes (BP), cellular components (CC), and molecular functions (MF), respectively. Differentially expressed lncRNA target genes are enriched in the cellular metabolic process and intracellular part (Figure 5a). Differentially expressed mRNA target genes were enriched in a single-organism metabolic process (Figure 5b).

### 2.7. KEGG Signaling Pathway Enrichment Analysis

**Figure 6:**
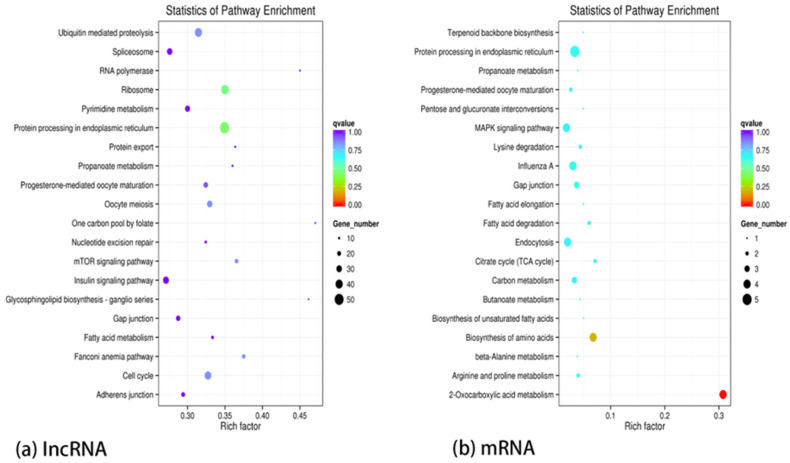
(**a**) KEGG enrichment of differentially expressed lncRNA target gene; (**b**) KEGG enrichment of differentially expressed mRNA target gene.

KEGG pathway significance enrichment identifies the most prominent biochemical metabolic pathways and signaling pathways in which specific genes are involved. It can be seen that differentially expressed lncRNA target genes are mainly involved in protein processing in the endoplasmic reticulum, ribosome, ubiquitin-mediated proteolysis, and oocyte meiosis (Figure 6a). The differentially expressed mRNA target genes are mainly involved in 2-oxocarboxylic acid metabolism, biosynthesis of amino acids, protein processing in endoplasmic reticulum, citrate cycle, and fatty acid degradation (Figure 6b). Among them, the target genes related to the regulation of heat stress were mainly enriched in the protein processing in endoplasmic reticulum pathways, which yielded genes such as *HSPA2*, *HSPH1*, *HSP90AA1*, and *HSPA8* (Table 3 and Table 4).

### 2.8. Expression Correlation Prediction of lncRNA and mRNA

Filtering of mRNAs expressing genes of the heat shock protein family showed that when *HSPA8*, *HSPH1*, and *HSPA2* of the HSP70 family and *DNAJA4* of the HSP40 family were expressed, they were accompanied by recurring lncRNAs such as *XLOC010450*, *XLOC037987*, *XLOC053511*, *XLOC061207*, and *XLOC100318* and that all of these genes are shown to be down-regulated (Table 5).

### 2.9. qPCR Validation

**Figure 7:**
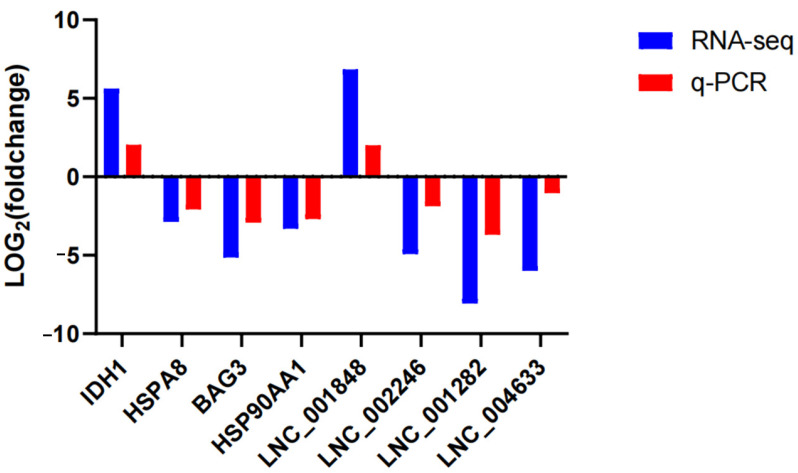
Validation of the differentially expressed genes by RT-PCR. *IDH1, HSPA8, BAG3,* and *HSP90AA1* are the differentially expressed mRNAs. *LNC_001282*, *LNC_001848*, *LNC_004633*, and *LNC_002246* are the differentially expressed lncRNAs.

To verify the reliability of the RNA sequencing data, four differentially expressed lncRNAs and four differentially expressed mRNAs were randomly selected for real-time quantitative PCR, and the quantitative results were basically consistent with the sequencing results (Figure 7).

## 3. Discussion

Heat stress has negative effects on the physiology, health, production, welfare, and behavior of poultry. Under heat stress, poultry not only undergo a series of physiological changes such as oxidative stress, acid-base imbalance, and immunosuppression [18], but also experience impaired reproductive function [19,20]. High-temperature environments can reduce exercise, increase water intake, rest periods, and intestinal microecological imbalances in poultry [21]. And it also decreases total serum antioxidant capacity and increases adenosine diphosphate (ADP) and adenosine monophosphate (AMP) levels in the liver of broilers [22]. It also increased fatty acid synthesis and the expression of fatty acid synthesis-related genes while reducing feed conversion, liver weight, and liver index in heat-stressed broilers [23].

Heat-stressed laying hens have reduced egg-laying performance due to damage to the follicular granular layer and impaired steroidogenesis [24]. Similar results have been reported by our laboratory in previous experiments [13]. Hepatic microvesicular lipid degradation and reduced glutamine synthetase activity occurred when broilers were exposed to high temperatures for 7 days, and hepatic vesicle degeneration and apoptosis occurred when exposed to high temperatures for 14 days [25]. Heat stress decreased serum levels of E2 and P4 in laying hens and reduced egg production by decreasing follicle numbers [26]. For this reason, researchers have also worked on adding functional nutrients to diets to alleviate heat stress in poultry. The addition of appropriate amounts of curcumin to poultry diets can improve immunity, antioxidant and antimicrobial activity, and resistance to heat stress [27]. Some studies have shown that curcumin can increase feed conversion, mitochondrial membrane potential, and sulfhydryl concentration and decrease serum aspartate and alanine aminotransferase activities in broilers [28]. Curcumin has been widely used as a dietary supplement to ameliorate the adverse effects of heat stress in poultry. Cardiac troponin Ι and angiotensin II were reduced in curcumin-treated mice, which exerted a cardioprotective effect on the mice’s heart [12], it also alleviates heat stress in quail by inhibiting oxidative stress and modulating the Nrf2/HO-1 pathway [29], and curcumin can also increase total antioxidant capacity, catalase, and glutathione peroxidase activities in laying hens [30]. The beneficial effects of curcumin have also been observed in our laboratory to significantly improve the decline in egg production, egg laying performance, and egg quality due to heat stress [13], even if we do not find out what its underlying mechanisms are.

The liver is a critical junction for many physiological processes, notably macronutrient metabolism, blood volume regulation, immune system support, endocrine control of growth signaling pathways, lipid and cholesterol homeostasis, and catabolism of exogenous compounds [31]. Heat stress decreases liver weight and leads to changes in the expression of key hepatic proteins involved in the heat shock response, immune defense, oxidative stress response, and apoptosis [32]. In this study, liver tissues of three laying hens from each of the 0 mg/kg and 150 mg/kg curcumin addition groups under heat stress were selected for RNA-Seq to ensure the reliability and accuracy of transcriptome analysis.

Alternative splicing is a process in which RNA exons produced by transcription of genes or pre-RNA are reconnected by RNA splicing in a variety of ways, and alternative splicing is the key to increasing protein complexity [33]. In a previous study, seven types of alternative splicing had been identified, including skipped exon, alternative 5′ splice site, alternative 3′splice site, mutually exclusive exons, retained intron, alternative promoter, and alternative polyadenylation. In our study, five types of alternative splicing were recognized from the data of sample RNA-seq, including skipped exon, alternative 5′ splice site, alternative 3′splice site, mutually exclusive exons, and retained intron. In earlier studies, it was found that heat stress caused the alternative 5′ splice site selection of *HSP47* in mice [34], and heat stress induces activation of the alternative 5′ splice site in the human genome [35]. In this study, the least type of Alternative splicing is the alternative 5′ splice site.

Previous studies have shown that lncRNAs interact with membrane-plasmic structures such as the endoplasmic reticulum and mitochondria and can regulate processes such as mRNA transport, stability, and translation [36]. It has been reported that some lncRNAs are co-expressed with the corresponding coding mRNAs, and co-expression of lncRNAs with mRNAs has been applied in nasopharyngeal, ovarian, acute cerebral hemorrhage, and bladder cancers to predict the progression of the disease and the prognosis, and to provide a genetic target for treatment [37,38,39,40]. This experiment also predicted target genes associated with heat stress by analyzing the correlation between lncRNA and mRNA expression.

HSP70 and HSP90 can maintain protein homeostasis and regulate cellular response to environmental stresses, and proteins promote HSP70-HSP90 interaction in the presence of ATP [41]. HSP70 protects cells by assisting protein refolding and stabilization, and the HSP70 gene was significantly up-regulated in *Sebastiscus marmoratus* after heat treatment [42]. Mongolian gerbils resisted heat stress by increasing HSP70 expression, and HSP70 expression was tissue/organ-specific, generally regulating HSP70 expression in the liver to alleviate heat stress [43]. The expression of HSP70, HSP60, and HSP47 in the intestine of poultry was significantly increased under heat stress, and coenzyme Q10 was found to increase the expression of HSP70 to protect chicken cardiomyocytes [44,45]. *HSPA8,* also called HSC70, a key chaperone within healthy cells and holds a significant role in facilitating protein transport, mediating endocytosis, orchestrating antigen processing and presentation, and catalyzing immune responses triggered by viruses [46,47,48]. As the first amyloidase in mammalian cells, *HSPA8* also can significantly inhibit necroptosis [49]. Previous studies have found that *HSPH1* can fight tumors by activating CD8+T cells [50,51]. *HSPA2* is a testis-enriched chaperone, which garnered attention as a significant protein in cancer with potential biomarker significance [52]. It is reported that *DNAJA4* can control the expression of F-actin and correlated pathway proteins in response to high temperature in HaCAT cells [53]. Also, it is an SREBP-regulated chaperone engaged in the cholesterol biosynthesis pathway [54]. In this study, *HSPA8*, *HSPH1*, *HSPA2,* and *DNAJA4* were down-regulated in the livers of laying hens in the experimental group with the addition of curcumin. This suggested that curcumin can alleviate heat stress in laying hens by down-regulating *HSPA8*, *HSPH1*, *HSPA2* and *DNAJA4* in the liver.

We screened mRNAs involved in the transcription and translation of genes of the heat shock protein family and found that when *HSPA8*, *HSPH1*, and *HSPA2* of the HSP70 family, and *DNAJA4* of the HSP40 family were expressed, lncRNAs such as *XLOC010450*, *XLOC037987*, *XLOC053511*, *XLOC061207*, and *XLOC100318* were accompanied by the recurring appearance of the corresponding mRNAs. Therefore, it can be concluded that several target genes of the above lncRNAs are associated with heat stress, and these lncRNAs co-expressed with mRNAs showed down-regulation of the genes *HSPA8*, *HSPH1*, *HSPA2*, and *DNAJA4* associated with heat stress. It shows that in the presence of curcumin, heat-stressed laying hens do not need much heat shock protein to resist heat stress, and it also shows that curcumin can alleviate heat stress in laying hens. In a previous study, transcriptome analysis of lncRNA in the hypothalamic-pituitary-mammary axis of dairy cows and liver tissues of Hu Sheep under heat stress revealed that the differentially expressed lncRNA target genes in the mammary gland of dairy cows were mainly enriched in apoptosis and AMPK and that differentially expressed mRNA in the liver of Hu Sheep was mainly enriched in PPAR and other signaling pathways related to heat stress [55,56]. In our study, the differentially expressed lncRNA target genes were mainly enriched in cellular metabolic processes and intracellular parts of the GO Pathway. *HSPA8*, *HSPH1*, and *HSPA2* were mainly enriched in the Protein processing in the endoplasmic reticulum KEGG Pathway and showed down-regulation.

## 4. Materials and Methods

### 4.1. Experimental Design and Sample Collect

The curcumin used in this study was provided by the Kehu Bio-technology Research Center (Guangzhou, China). The content of curcumin was 98% in this study [13]. A group of 240 Hy-Line brown hens, which were aged 280 days in the peak laying stage, were distributed randomly into four different experimental diets. Each diet had four replicates, and each replicate consisted of 15 birds. The hens were reared under the same condition and kept in separate cages. A few amounts of the basal diet were firstly mixed with an appropriate amount of curcumin to make a small batch, and then the remaining basal diet was added to obtain a well-homogeneous mixture [13]. All hens were given unrestricted access to fresh water and feed, while indoor ventilation and lighting were ensured. Additionally, their living environment underwent routine cleaning and disinfection. The photoperiod was programmed for 16 h of light and 8 h of darkness. We measured the temperature of the house at 9:00 a.m., 13:00 p.m., 17:00 p.m., and 21:00 p.m. every day, and the ambient temperature was adjusted to 34 ± 2 °C for 8 h per day (9:00 a.m.–5:00 p.m.), transitioning to a range of 22 °C to 28 °C for the remaining 16 h. The feeding experiment was conducted for 42 days after a 10-day adaptation period [13]. Previous experiments in our laboratory confirmed that diet supplementation with 150 mg/kg of curcumin exhibited the best antioxidant capability and production performance. The hens were executed by cervical dislocation, followed by placing the carcasses on the autopsy table. Liver tissue samples were collected from the whole liver of each hen, three liver tissues from each of 0 mg/kg and 150 mg/kg curcumin test groups were collected, resulting in a total of six samples. These samples were promptly frozen using liquid nitrogen and stored at a temperature of −80 °C for preservation. The basal diet was formulated to meet or exceed the National Research Council (NRC, 1994) recommendations. All animal experiments were approved by the Animal Care and Use Committee of Nanjing Agricultural University (Nanjing, China).

### 4.2. RNA Extraction, Library Construction, Quality Control, and RNA-seq

The total RNA was extracted from each sample by RNAiso Plus (TaKaRa, Kyoto, Japan) and recovered into a 15 uL elution volume using ddH2O containing one thousandth of DEPC. The RNA extraction procedure was carried out according to the manufacturer’s guidelines provided with the RNAiso Plus. The concentration and purity of RNA were determined by the NanoDrop spectrophotometer (Thermo Fisher Scientific, Waltham, MA, USA), and RNA quality was accurately detected with Agilent 2100 bioanalyzer (Agilent Technologies, Palo Alto, CA, USA). Samples that passed the quality assessment test were sent to Allwegene Technology (Allwegene Technology Co., Ltd., Beijing, China) for the transcriptome sequencing test. The construction of the lncRNA library is strand-specific; the initial step of reverse transcription synthesis for the first cDNA chain is identical to the NEB’s ordinary library construction method. However, there was a difference that dTTP in dNTPs was replaced by dUTP when the second chain was being synthesized. Subsequent steps include end repair, A tailing, ligate to U-adaptor, and length screening, which are carried out conventionally. Finally, the second cDNA chain containing dUTP was degraded using the USER enzyme, followed by PCR amplification to generate the library [57,58]. After the construction of the library, Qubit 2.0 was used for preliminary quantification, and then the insert size of the library was detected by the Agilent Bioanalyzer 2100 system. qPCR was used to accurately quantify the effective concentration of the library. After assessing the quality of the library, the six libraries were sequenced on Illumina HiSeq™ 2000 from Beijing Allwegene Co., Ltd., (Beijing, China).

### 4.3. Reads Alignments and Assembled Transcripts

The filtered reads were mapped to the chicken reference genome (Gallus_gallus-4.0, ensemble database) using HISAT2 (Version: 2.0.4). In the absence of contamination and with appropriate reference genes, the percentage of aligned reads produced by the sequencing of the experimental sample was higher than 70%. Junction count only and Reads on target and junction counts were used to count the type and amount of alternative splicing that occurred in RNA-seq data [59]. Subsequently, the transcriptome assembly of the HISAT2 (Version: 2.0.4) alignment results was conducted using StringTie (Version: 1.3.1), followed by quantitative analysis of the transcripts [60].

### 4.4. LncRNA Filtration and Inter-Sample Correlation Detection

The cuffmerge program of cufflinks (Version: 2.0) was used to assemble transcripts obtained from each sample, and transcripts with uncertain chain orientation were removed; this process yields comprehensive transcriptome information for this sequencing session [61]. Subsequently, the assembled transcript set was subjected to lncRNA filtration. By integrating various mainstream methods for coding potential analysis, transcripts identified as lacking coding potential across these analyses are selected as the predicted dataset of lncRNAs for this study. We used the Pearson correlation coefficient to assess the correlation of expression between samples. The closer the correlation coefficient is to 1, the higher the similarity in expression patterns between samples. In our specific project, we required a Pearson correlation coefficient of at least 0.8. Then, transcripts were identified using CPC2 [62], PFAM [63], PhyloCSF [64], and CNCI [65] and compared with the results predicted by the cuffmerge program.

### 4.5. Identification of Differential Expressed Genes and Analysis of GO Category and KEGG Pathway

We employed edgeR (Version: 3.0.8) to conduct differential analysis across various types of transcripts. Generating a volcano plot visually depicts the overall distribution of differentially expressed transcripts or genes. The threshold for screening was set as qvalue < 0.05 by default. We used cluster analysis to determine the expression patterns of differentially expressed transcripts under different experimental conditions. Hierarchical clustering analysis was performed based on FPKM (Fragments Per Kilobase of exon model per Million mapped fragments) values of differentially expressed transcripts across various experimental conditions. After screening the differential genes according to the experimental purpose, the GO annotation information of all genes was extracted from the Gene Ontology database, and then GO enrichment analysis was performed using the GOseq software (Version: 1.22), and the GO terms of the differential genes with a corrected P-value of less than 0.05 were selected as significantly enriched [66], and the association of differential genes with different pathways was analyzed using KOBAS (Version: 2.0) with a corrected *p*-value of 0.05 as the threshold for identifying significantly different pathways [67,68].

### 4.6. Quantitative Real-Time PCR (qRT-PCR) Validation

To verify the reliability of the RNA-seq results, four mRNAs (*IDH1*, *BAG3*, *HSP90AA1*, *HSPA8*) and four lncRNAs (*LNC_001282*, *LNC_001848*, *LNC_004633*, *LNC_002246*) were randomly selected for quantitative real-time PCR. Total RNA was extracted from liver tissue samples using RNAios plus (TaKaRa, Kyoto, Japan). The NanoDrop spectrophotometer (Thermo Fisher Scientific, Waltham, MA, USA) was used for determining the concentration of the RNA. Total RNA was reverse transcribed by using the Primescript RT Master Mix (TaKaRa, Kyoto, Japan). RNA was reverse transcribed into cDNA by using the AidTM First Strand cDNA Synthesis Kit (Thermo Fisher Scientific, Waltham, MA USA). To design the primers with the Primer3 Input (Version: 0.4.0, http://bioinfo.ut.ee/primer3-0.4.0/, (accessed on 15 November 2023)) online. The primers were synthesized by Shanghai Sangon Co., Ltd. (Shanghai, China) (Table 6). Each reaction of RT-PCR was in the volume of 20 μL, containing 10 μL the SYBR Green mix, 2 μL cDNA, and 0.2 μM each primer. Each sample was analyzed in triplicate, and negative controls were included in all qPCR runs. The relative expression of each mRNA was estimated using the 2^−ΔΔCT^ method [69].

## 5. Conclusions

This study provides a basis for learning about transcriptional changes in the liver of laying hens and the effect of dietary supplementation with curcumin on the transcriptional changes in lncRNA in the liver of laying hens under heat stress conditions. High-throughput sequencing of the livers of laying hens fed curcumin predicted the heat stress-related genes that mRNAs co-expressing with lncRNAs, such as *HSPA8*, *HSPH1*, *HSPA2*, and *DNAJA4*, and found that all of these heat stress-related genes were down-regulated. These data suggested that curcumin may alleviate heat stress in laying hens by down-regulating the liver genes of heat-stress-related protein such as *HSPA8*, *HSPH1*, etc. And laid the groundwork for understanding the underlying mechanisms of the heat stress response, as well as the potential benefits of curcumin supplementation of laying hens’ diets under heat stress conditions.

## Figures and Tables

**Table 1:** Summary of the RNA-seq result of liver tissue samples from the control group and curcumin treat group.

Sample Name	Raw Reads	Clean Reads	Clean Bases	Q20 (%)	Q30 (%)	GC Content (%)
CTR1	141,354,366	138,329,490	20.75 G	97.07	92.86	48.87
CTR2	123,877,456	121,554,156	18.23 G	97.09	92.91	51.66
CTR3	144,998,210	142,024,464	21.3 G	97.18	93.11	48.06
Treat1	126,852,994	125,635,340	18.85 G	97.55	93.61	49.68
Treat2	205,193,728	201,015,402	30.15 G	97.19	93.13	50.07
Treat3	119,451,560	117,319,078	17.6 G	97.29	93.34	49.88

Q20, Q30: the ratio of the number of bases with a Phred quality value greater than 20, 30 to the total number of bases (clean data).

**Table 2:** Result of clean reads from six samples mapped to reference genome.

Sample Name	Total Reads	Total Mapped	Multiple Mapped	Uniquely Mapped
CTR1	138,329,490	129,787,959 (93.83%)	14,303,032 (10.34%)	115,484,927 (83.49%)
CTR2	121,554,156	113,126,334 (93.07%)	15,063,522 (12.39%)	98,062,812 (80.67%)
CTR3	142,024,464	134,005,785 (94.35%)	13,746,535 (9.68%)	120,259,250 (84.68%)
Treat1	60,167,536	56,707,140 (94.25%)	7,168,028 (11.91%)	49,539,112 (82.34%)
Treat2	201,015,402	188,897,443 (93.97%)	22,901,129 (11.39%)	165,996,314 (82.58%)
Treat3	117,319,078	109,761,781 (93.56%)	14,668,105 (12.5%)	95,093,676 (81.06%)

Total reads: the total number of reads after quality control. Total mapped: the total number of reads that can be compared to the reference sequence. Multiple mapped: the number of reads that are compared to multiple positions in the reference sequence. Uniquely mapped: for the number of reads at the unique position of the reference sequence. Reads map to ‘+’, reads map to ‘−’: align the number of Reads to the positive and negative strands of the sequence, respectively. Splice reads: align the same read segment to different outside non-splice reads: the total number of the same read only aligned to one exon.

**Table 3:** KEGG pathways of differentially expressed lncRNA target gene.

KEGG Pathway ID	Term	Cg	Bg	Gene Name
gga04141	Protein processing in endoplasmic reticulum	5	146	*|HSPA2, HSP70|HSPH1|HSP90AA1|HSPA8, HS* *C* *70|*
gga03010	Ribosome	1	120	*RPL7A*
gga04120	Ubiquitin mediated proteolysis	1	124	*CUL5*
gga04114	Oocyte meiosis	1	88	*ADCY1*

**Table 4:** KEGG Pathways of differentially expressed mRNA target gene.

KEGG Pathway ID	Term	Cg	Bg	Gene Name
gga01210	2-Oxocarboxylic acid metabolism	4	13	*|ACY1|IDH3B|IDH1|ACY1|*
gga01230	Biosynthesis of amino acids	4	59	*|ACY1|IDH3B|IDH1|ACY1|*
gga04141	Protein processing in endoplasmic reticulum	5	146	*|HSPA2, HSP70|HSPH1|HSP90AA1|HSPA8, HSC70|*
gga00020	Citrate cycle (TCA cycle)	2	28	*|IDH1|IDH3B|*
gga00071	Fatty acid degradation	2	33	*|HADHA|ECI1, DCI|*

KEGG pathway ID is unique numbering information for pathways in the KEGG database; Term is KEGG Pathway Description Information; Cg is the number of differential lncRNA target genes or differentially expressed mRNA counterparts in the pathway; Bg is the number of genes under the pathway; Gene name is the name of genes enriched in the KEGG pathway.

**Table 5:** LncRNA co-expression mRNA prediction.

mRNA Gene Symbol	mRNA Gene ID	LncRNA Gene ID	Mrna Gene Description	Control Group vs. Curcumin Addition Group
*HSPA8*	ENSGALG00000006512	*XLOC_053511 XLOC_037987 XLOC_010450 XLOC_100318 XLOC_061207*	Heat shock protein 70 family||Heat shock protein 70 kD, peptide-binding domain||Heat shock protein 70, conserved site||Heat shock protein 70 kD, C-terminal domain	Down
*HSPH1*	ENSGALG00000017077	*XLOC_061207 XLOC_053511 XLOC_037987 XLOC_010450*	Heat shock protein 70 family||Heat shock protein 70 kD, C-terminal domain||Heat shock protein 70, conserved site||Heat shock protein 70 kD, peptide-binding domain	Down
*HSPA2*	ENSGALG00000011715	*XLOC_100318 XLOC_061207 XLOC_037987*	Heat shock protein 70 kD, C-terminal domain||Heat shock protein 70 kD, peptide-binding domain||Heat shock protein 70, conserved site||Heat shock protein 70 family	Down
*DNAJA4*	ENSGALG00000036850	*XLOC_061207 XLOC_037987 XLOC_053511*	HSP40/DnaJ peptide-binding||Chaperone DnaJ, C-terminal||DnaJ domain||DnaJ domain, conserved site||Chaperone DnaJ||Heat shock protein DnaJ, cysteine-rich domain	Down

**Table 6:** Primers of genes used in RT-qPCR.

Gene ID	Transcript ID	Primer Sequence	Product Length (bp)
*IDH1*	ENSGALT00000014333	F: CCCCATTGCCTCCATCTTTG R: CAAGCAGCAAGGTCCTTTGT	153
*HSPA8*	ENSGALT00000086458	F: AGCGACAGGGCACAAAAGAT R: GGTTCCTTTCAGCACCAACTTTC	123
*BAG3*	ENSGALT00000088658	F: TTCGGTCACCTACCGAGAAC R: CCCAGTTTCTTCAGGAGCAG	139
*HSP90AA1*	ENSGALT00000081765	F: CAAGCCTATTTGGACCAGGA R: GCTCTGAATTCCAGCTGACC	138
*XLOC_015361*	LNC_001282	F: CCGCTTTTCGCCCCAAAATGAG R: CAGACCCATAGATTGACCCATGAGG	130
*XLOC_027753*	LNC_001848	F: AGGCACACTCTAGAAAGGCAC R: CATCCCCCAACTTCTCGTTGA	122
*XLOC_097982*	LNC_004633	F: GCATTAGAGCTGCACCAACA R: TTGCCATGGTTCATTTCTCA	150
*XLOC_037987*	LNC_002246	F: TGTGATGGTTTACTGCCACC R: AGCCCGTGTAAGGTCTAAAGTG	127

Gene ID is the name of the gene; Transcript ID is the name of the Transcript; Primer Sequence is the sequences of the primer.

## Data Availability

Date is contained within the article.

## References

[1] Kim D.-H., Lee K.-W. (2023). An Update on Heat Stress in Laying Hens. World’s Poult. Sci. J..

[2] Altan O., Pabuçcuoğlu A., Altan A., Konyalioğlu S., Bayraktar H. (2003). Effect of Heat Stress on Oxidative Stress, Lipid Peroxidation and Some Stress Parameters in Broilers. Br. Poult. Sci..

[3] Lin H., Decuypere E., Buyse J. (2006). Acute Heat Stress Induces Oxidative Stress in Broiler Chickens. Comp. Biochem. Physiol. A. Mol. Integr. Physiol..

[4] Shini S., Huff G.R., Shini A., Kaiser P. (2010). Understanding Stress-Induced Immunosuppression: Exploration of Cytokine and Chemokine Gene Profiles in Chicken Peripheral Leukocytes. Poult. Sci..

[5] Sahin K., Sahin N., Kucuk O., Hayirli A., Prasad A.S. (2009). Role of Dietary Zinc in Heat-Stressed Poultry: A Review. Poult. Sci..

[6] Lin H., Jiao H.C., Buyse J., Decuypere E. (2006). Strategies for Preventing Heat Stress in Poultry. World’s Poult. Sci. J..

[7] Roushdy E.M., Zaglool A.W., El-Tarabany M.S. (2018). Effects of Chronic Thermal Stress on Growth Performance, Carcass Traits, Antioxidant Indices and the Expression of HSP70, Growth Hormone and Superoxide Dismutase Genes in Two Broiler Strains. J. Therm. Biol..

[8] Kim D.Y., Lim B., Kim J.M., Kil D.Y. (2022). Integrated Transcriptome Analysis for the Hepatic and Jejunal Mucosa Tissues of Broiler Chickens Raised under Heat Stress Conditions. J. Anim. Sci. Biotechnol..

[9] Patel S.S., Acharya A., Ray R.S., Agrawal R., Raghuwanshi R., Jain P. (2020). Cellular and Molecular Mechanisms of Curcumin in Prevention and Treatment of Disease. Crit. Rev. Food Sci. Nutr..

[10] Aggarwal B.B., Gupta S.C., Sung B. (2013). Curcumin: An Orally Bioavailable Blocker of TNF and Other pro-Inflammatory Biomarkers. Br. J. Pharmacol..

[11] Nawab A., Li G., An L., Wu J., Chao L., Xiao M., Zhao Y., Birmani M.W., Ghani M.W. (2019). Effect of Curcumin Supplementation on TLR4 Mediated Non-Specific Immune Responses in Liver of Laying Hens under High-Temperature Conditions. J. Therm. Biol..

[12] Chen Y., Jiang W., Liu X., Du Y., Liu L., Ordovas J.M., Lai C.-Q., Shen L. (2020). Curcumin Supplementation Improves Heat-Stress-Induced Cardiac Injury of Mice: Physiological and Molecular Mechanisms. J. Nutr. Biochem..

[13] Liu M., Lu Y., Gao P., Xie X., Li D., Yu D., Yu M. (2020). Effect of Curcumin on Laying Performance, Egg Quality, Endocrine Hormones, and Immune Activity in Heat-Stressed Hens. Poult. Sci..

[14] Wu Z., Liu X., Liu L., Deng H., Zhang J., Xu Q., Cen B., Ji A. (2014). Regulation of lncRNA Expression. Cell. Mol. Biol. Lett..

[15] Muret K., Klopp C., Wucher V., Esquerré D., Legeai F., Lecerf F., Désert C., Boutin M., Jehl F., Acloque H. (2017). Long Noncoding RNA Repertoire in Chicken Liver and Adipose Tissue. Genet. Sel. Evol..

[16] Li Q., Qiao J., Zhang Z., Shang X., Chu Z., Fu Y., Chu M. (2020). Identification and Analysis of Differentially Expressed Long Non-Coding RNAs of Chinese Holstein Cattle Responses to Heat Stress. Anim. Biotechnol..

[17] Dou J., Schenkel F., Hu L., Khan A., Khan M.Z., Yu Y., Wang Y., Wang Y. (2021). Genome-Wide Identification and Functional Prediction of Long Non-Coding RNAs in Sprague-Dawley Rats during Heat Stress. BMC Genom..

[18] Onagbesan O.M., Uyanga V.A., Oso O., Tona K., Oke O.E. (2023). Alleviating Heat Stress Effects in Poultry: Updates on Methods and Mechanisms of Actions. Front. Vet. Sci..

[19] Rozenboim I., Tako E., Gal-Garber O., Proudman J.A., Uni Z. (2007). The Effect of Heat Stress on Ovarian Function of Laying Hens. Poult. Sci..

[20] Ebeid T.A., Suzuki T., Sugiyama T. (2012). High Ambient Temperature Influences Eggshell Quality and Calbindin-D28k Localization of Eggshell Gland and All Intestinal Segments of Laying Hens. Poult. Sci..

[21] Lara L.J., Rostagno M.H. (2013). Impact of Heat Stress on Poultry Production. Animals.

[22] Ahmed-Farid O., Salah A.S., Nassan M.A., El-Tarabany M.S. (2021). Effects of Chronic Thermal Stress on Performance, Energy Metabolism, Antioxidant Activity, Brain Serotonin, and Blood Biochemical Indices of Broiler Chickens. Animals.

[23] Tang L.-P., Liu Y.-L., Zhang J.-X., Ding K.-N., Lu M.-H., He Y.-M. (2022). Heat Stress in Broilers of Liver Injury Effects of Heat Stress on Oxidative Stress and Autophagy in Liver of Broilers. Poult. Sci..

[24] Yan L., Hu M., Gu L., Lei M., Chen Z., Zhu H., Chen R. (2022). Effect of Heat Stress on Egg Production, Steroid Hormone Synthesis, and Related Gene Expression in Chicken Preovulatory Follicular Granulosa Cells. Animals.

[25] Ma B., Xing T., Li J., Zhang L., Jiang Y., Gao F. (2022). Chronic Heat Stress Causes Liver Damage via Endoplasmic Reticulum Stress-Induced Apoptosis in Broilers. Poult. Sci..

[26] Li G.-M., Liu L.-P., Yin B., Liu Y.-Y., Dong W.-W., Gong S., Zhang J., Tan J.-H. (2020). Heat Stress Decreases Egg Production of Laying Hens by Inducing Apoptosis of Follicular Cells via Activating the FasL/Fas and TNF-α Systems. Poult. Sci..

[27] Moniruzzaman M., Min T. (2020). Curcumin, Curcumin Nanoparticles and Curcumin Nanospheres: A Review on Their Pharmacodynamics Based on Monogastric Farm Animal, Poultry and Fish Nutrition. Pharmaceutics.

[28] Zhang J., Bai K.W., He J., Niu Y., Lu Y., Zhang L., Wang T. (2018). Curcumin Attenuates Hepatic Mitochondrial Dysfunction through the Maintenance of Thiol Pool, Inhibition of mtDNA Damage, and Stimulation of the Mitochondrial Thioredoxin System in Heat-Stressed Broilers. J. Anim. Sci..

[29] Sahin K., Orhan C., Tuzcu Z., Tuzcu M., Sahin N. (2012). Curcumin Ameloriates Heat Stress via Inhibition of Oxidative Stress and Modulation of Nrf2/HO-1 Pathway in Quail. Food Chem. Toxicol..

[30] Nawab A., Li G., Liu W., Lan R., Wu J., Zhao Y., Kang K., Kieser B., Sun C., Tang S. (2019). Effect of Dietary Curcumin On the Antioxidant Status of Laying Hens Under High-Temperature Conditions. Chem. Chem..

[31] Trefts E., Gannon M., Wasserman D.H. (2017). The Liver. Curr. Biol..

[32] Cui Y., Hao Y., Li J., Bao W., Li G., Gao Y., Gu X. (2016). Chronic Heat Stress Induces Immune Response, Oxidative Stress Response, and Apoptosis of Finishing Pig Liver: A Proteomic Approach. Int. J. Mol. Sci..

[33] Liu Q., Fang L., Wu C. (2022). Alternative Splicing and Isoforms: From Mechanisms to Diseases. Genes.

[34] Takechi H., Hosokawa N., Hirayoshi K., Nagata K. (1994). Alternative 5′ Splice Site Selection Induced by Heat Shock. Mol. Cell. Biol..

[35] Nevo Y., Kamhi E., Jacob-Hirsch J., Amariglio N., Rechavi G., Sperling J., Sperling R. (2012). Genome-Wide Activation of Latent Donor Splice Sites in Stress and Disease. Nucleic Acids Res..

[36] Herman A.B., Tsitsipatis D., Gorospe M. (2022). Integrated lncRNA Function upon Genomic and Epigenomic Regulation. Mol. Cell.

[37] Mao Y., Wen C., Yang Z. (2022). Construction of a Co-Expression Network for lncRNAs and mRNAs Related to Urothelial Carcinoma of the Bladder Progression. Front. Oncol..

[38] Lu X., Chen X., Wang X., Qing J., Li J., Pan Y. (2022). Construction of lncRNA and mRNA Co-Expression Network Associated with Nasopharyngeal Carcinoma Progression. Front. Oncol..

[39] Xiao Q., Luo J., Liang C., Li G., Cai J., Ding P., Liu Y. (2020). Identifying lncRNA and mRNA Co-Expression Modules from Matched Expression Data in Ovarian Cancer. IEEE/ACM Trans. Comput. Biol. Bioinf..

[40] Yu Z., Hu E., Cai Y., Zhu W., Chen Q., Li T., Li Z., Wang Y., Tang T. (2023). mRNA and lncRNA Co-Expression Network in Mice of Acute Intracerebral Hemorrhage. Front. Mol. Neurosci..

[41] Doyle S.M., Hoskins J.R., Kravats A.N., Heffner A.L., Garikapati S., Wickner S. (2019). Intermolecular Interactions between Hsp90 and Hsp70. J. Mol. Biol..

[42] Han X., Jin S., Shou C., Han Z. (2023). Hsp70 Gene Family in Sebastiscus Marmoratus: The Genome-Wide Identification and Transcriptome Analysis under Thermal Stress. Genes.

[43] Lou S.-L., Zhang X.-Y., Wang D.-H. (2019). HSP70 Plays a Role in the Defense of Acute and Chronic Heat Stress in Mongolian Gerbils (Meriones Unguiculatus). J. Therm. Biol..

[44] Al-Zghoul M.B. (2018). Thermal Manipulation during Broiler Chicken Embryogenesis Increases Basal mRNA Levels and Alters Production Dynamics of Heat Shock Proteins 70 and 60 and Heat Shock Factors 3 and 4 during Thermal Stress. Poult. Sci..

[45] Xu J., Tang S., Song E., Yin B., Wu D., Bao E. (2017). Hsp70 Expression Induced by Co-Enzyme Q10 Protected Chicken Myocardial Cells from Damage and Apoptosis under in Vitro Heat Stress. Poult. Sci..

[46] Hartl F.U., Bracher A., Hayer-Hartl M. (2011). Molecular Chaperones in Protein Folding and Proteostasis. Nature.

[47] Balchin D., Hayer-Hartl M., Hartl F.U. (2016). In Vivo Aspects of Protein Folding and Quality Control. Science.

[48] Bonam S.R., Ruff M., Muller S. (2019). HSPA8/HSC70 in Immune Disorders: A Molecular Rheostat That Adjusts Chaperone-Mediated Autophagy Substrates. Cells.

[49] Wu E., He W., Wu C., Chen Z., Zhou S., Wu X., Hu Z., Jia K., Pan J., Wang L. (2023). HSPA8 Acts as an Amyloidase to Suppress Necroptosis by Inhibiting and Reversing Functional Amyloid Formation. Cell Res..

[50] Miyazaki M., Nakatsura T., Yokomine K., Senju S., Monji M., Hosaka S., Komori H., Yoshitake Y., Motomura Y., Minohara M. (2005). DNA Vaccination of HSP105 Leads to Tumor Rejection of Colorectal Cancer and Melanoma in Mice through Activation of Both CD4 T Cells and CD8 T Cells. Cancer Sci..

[51] Shimizu Y., Yoshikawa T., Kojima T., Shoda K., Nosaka K., Mizuno S., Wada S., Fujimoto Y., Sasada T., Kohashi K. (2019). Heat Shock Protein 105 Peptide Vaccine Could Induce Antitumor Immune Reactions in a Phase I Clinical Trial. Cancer Sci..

[52] Scieglinska D., Sojka D.R., Gogler-Pigłowska A., Chumak V., Krawczyk Z. (2020). Various Anti-HSPA2 Antibodies Yield Different Results in Studies on Cancer-Related Functions of Heat Shock Protein A2. Int. J. Mol. Sci..

[53] Liu R.-J., Niu X.-L., Yuan J.-P., Chen H.-D., Gao X.-H., Qi R.-Q. (2020). DnaJA4 Is Involved in Responses to Hyperthermia by Regulating the Expression of F-Actin in HaCaT Cells. Chin. Med. J..

[54] Robichon C., Varret M., Le Liepvre X., Lasnier F., Hajduch E., Ferré P., Dugail I. (2006). DnaJA4 Is a SREBP-Regulated Chaperone Involved in the Cholesterol Biosynthesis Pathway. Biochim. Biophys. Acta.

[55] Li Y., Kong L., Deng M., Lian Z., Han Y., Sun B., Guo Y., Liu G., Liu D. (2019). Heat Stress-Responsive Transcriptome Analysis in the Liver Tissue of Hu Sheep. Genes.

[56] Li G., Yu X., Portela Fontoura A.B., Javaid A., De La Maza-Escolà V.S., Salandy N.S., Fubini S.L., Grilli E., McFadden J.W., Duan J.E. (2023). Transcriptomic Regulations of Heat Stress Response in the Liver of Lactating Dairy Cows. BMC Genom..

[57] Naphade S., Bhatnagar R., Hanson-Smith V., Choi I., Zhang A. (2022). Systematic Comparative Analysis of Strand-Specific RNA-Seq Library Preparation Methods for Low Input Samples. Sci. Rep..

[58] Mills J.D., Kawahara Y., Janitz M. (2013). Strand-Specific RNA-Seq Provides Greater Resolution of Transcriptome Profiling. Curr. Genom..

[59] Shen S., Park J.W., Lu Z., Lin L., Henry M.D., Wu Y.N., Zhou Q., Xing Y. (2014). rMATS: Robust and Flexible Detection of Differential Alternative Splicing from Replicate RNA-Seq Data. Proc. Natl. Acad. Sci. USA.

[60] Pertea M., Kim D., Pertea G.M., Leek J.T., Salzberg S.L. (2016). Transcript-Level Expression Analysis of RNA-Seq Experiments with HISAT, StringTie and Ballgown. Nat. Protoc..

[61] Pertea M., Pertea G.M., Antonescu C.M., Chang T.-C., Mendell J.T., Salzberg S.L. (2015). StringTie Enables Improved Reconstruction of a Transcriptome from RNA-Seq Reads. Nat. Biotechnol..

[62] Kang Y.-J., Yang D.-C., Kong L., Hou M., Meng Y.-Q., Wei L., Gao G. (2017). CPC2: A Fast and Accurate Coding Potential Calculator Based on Sequence Intrinsic Features. Nucleic Acids Res..

[63] Mistry J., Chuguransky S., Williams L., Qureshi M., Salazar G.A., Sonnhammer E.L.L., Tosatto S.C.E., Paladin L., Raj S., Richardson L.J. (2021). Pfam: The Protein Families Database in 2021. Nucleic Acids Res..

[64] Lin M.F., Jungreis I., Kellis M. (2011). PhyloCSF: A Comparative Genomics Method to Distinguish Protein Coding and Non-Coding Regions. Bioinformatics.

[65] Sun L., Luo H., Bu D., Zhao G., Yu K., Zhang C., Liu Y., Chen R., Zhao Y. (2013). Utilizing Sequence Intrinsic Composition to Classify Protein-Coding and Long Non-Coding Transcripts. Nucleic Acids Res..

[66] Young M.D., Wakefield M.J., Smyth G.K., Oshlack A. (2010). Gene Ontology Analysis for RNA-Seq: Accounting for Selection Bias. Genome Biol..

[67] Kanehisa M., Araki M., Goto S., Hattori M., Hirakawa M., Itoh M., Katayama T., Kawashima S., Okuda S., Tokimatsu T. (2008). KEGG for Linking Genomes to Life and the Environment. Nucleic Acids Res..

[68] Mao X., Cai T., Olyarchuk J.G., Wei L. (2005). Automated Genome Annotation and Pathway Identification Using the KEGG Orthology (KO) as a Controlled Vocabulary. Bioinformatics.

[69] Livak K.J., Schmittgen T.D. (2001). Analysis of Relative Gene Expression Data Using Real-Time Quantitative PCR and the 2(-Delta Delta C(T)) Method. Methods.

